# Prognostic subgroups of chronic pain patients using latent variable mixture modeling within a supervised machine learning framework

**DOI:** 10.1038/s41598-024-62542-w

**Published:** 2024-05-31

**Authors:** Xiang Zhao, Katharina Dannenberg, Dirk Repsilber, Björn Gerdle, Peter Molander, Hugo Hesser

**Affiliations:** 1 School of Behavioural, Social and Legal Sciences, Örebro University, Fakultetsgatan 1, 702 81 Örebro, Sweden; 2 School of Medical Sciences, Örebro University, Örebro, Sweden; 3 Department of Health, Medicine and Caring Sciences, Pain and Rehabilitation Centre, Linköping University, Linköping, Sweden; 4 Department of Behavioural Sciences and Learning, Linköping University, Linköping, Sweden

**Keywords:** Pain classification, Latent variable mixture modeling, Machine learning, Pain prognosis, Prognosis, Chronic pain

## Abstract

The present study combined a supervised machine learning framework with an unsupervised method, finite mixture modeling, to identify prognostically meaningful subgroups of diverse chronic pain patients undergoing interdisciplinary treatment. Questionnaire data collected at pre-treatment and 1-year follow up from 11,995 patients from the Swedish Quality Registry for Pain Rehabilitation were used. Indicators measuring pain characteristics, psychological aspects, and social functioning and general health status were used to form subgroups, and pain interference at follow-up was used for the selection and the performance evaluation of models. A nested cross-validation procedure was used for determining the number of classes (inner cross-validation) and the prediction accuracy of the selected model among unseen cases (outer cross-validation). A four-class solution was identified as the optimal model. Identified subgroups were separable on indicators, predictive of long-term outcomes, and related to background characteristics. Results are discussed in relation to previous clustering attempts of patients with diverse chronic pain conditions. Our analytical approach, as the first to combine mixture modeling with supervised, targeted learning, provides a promising framework that can be further extended and optimized for improving accurate prognosis in pain treatment and identifying clinically meaningful subgroups among chronic pain patients.

## Introduction

Subgrouping of patients with chronic pain has been an enduring research topic as it provides information for clinical assessment, accurate prognosis, and personalized healthcare^1,2^. The negative outcomes of chronic pain—pain that has lasted over three months—include disability, poor quality of life, and high health care consumption^3,4^. Clinical characteristics, presentation, and functioning among patients suggest significant individual heterogeneities^2,5–8^. Given that multiple biopsychosocial factors are known to contribute to chronic pain, interdisciplinary treatment is commonly the treatment of choice for most patients^4,9^. Yet, while findings indicate that patients with the severest clinical presentation seem to benefit most from such a treatment^10^, it is still unclear which patient- and treatment-related factors contribute to the treatment success^11^.

Data-driven approaches use large-scale datasets and rich information for decision making, providing a unique opportunity to identify patterns of information that can be used to improve classification and prediction in clinical health research^12^. Given the notable heterogeneity in both clinical presentation and treatment response, several studies have also been devoted to identifying clinically relevant subgroups using data-driven approaches in specific pain diagnoses^13,14^.

Although it is increasingly recognized that patients with a certain chronic pain diagnosis are heterogenous in clinical presentation^8^, less attention has been paid to identifying subgroups among a broader population of chronic pain patients. Yet, a handful of studies have examined subgroups in patients receiving interdisciplinary treatment, providing initial evidence for clinically relevant subgroups across diverse pain conditions^10,15–17^.

There are two major drawbacks of the current pain classification research. First, psychological and social dimensions are not well incorporated into the classification processes, even though a substantial body of evidence suggests that psychological and social aspects are important for the development, maintenance and treatment of pain^4,18,19^. Clustering attempts (i.e., identifying subgroups) have either been focused on specific psychological or biomedical aspects of pain, with only few studies having combined psychosocial dimensions and pain characteristics (e.g., pain extent, pain intensity)^20^. Second, the analytical approach to identifying subgroups has a large space to advance. Specifically, early subgroup profiling attempts used a limited number of indicator variables to form subgroups or primitive subgrouping methods (e.g., by using arbitrary cut-off points on a single measure or by using simple regression models). Thus, better categorization attempts are needed especially to improve the prediction of response to treatment and the matching of patients to treatments^21^.

In contrast to these early subgroup identifying approaches, advanced data-driven analytical methods aiming to uncover subgroups in heterogenous populations may hold more promise. Latent variable mixture modeling (LVMM) is an advanced person-centered analytic method, which enables researchers to classify individuals into unobserved (latent) subpopulations based on patterns of individual characteristics^22,23^ and may offer valuable information for individual level empirical classification and prediction^16,24^. Although underutilized in pain research overall the method has more recently gained increased attention in the field^24^.

While LVMM is a promising method, there are also some key analytical challenges when using LVMM, like any other data-driven approach, for the purpose of identifying clinically meaningful subgroups in a heterogenous population, such as chronic pain patients. In the applied sciences, LVMM is frequently used in an exploratory manner, with model selection relying primarily on comparing several models in terms of their quality of fit. Overfitting is the primary concern with any exploratory model, and this is also true for LVMM. Furthermore, no single criterion can be used to determine optimal fit, so researchers will have to rely on a combination of statistical and pragmatic criteria to select a final model. In fact, derived subgroups from LVMM have been difficult to replicate in independent samples and, given the considerable uncertainty over model selection, a general recommendation is not to treat the “best-fitted model” for the sample as the only model for the population^23^. Furthermore, methodologists have emphasized the necessity of “substantive checking”, such as linking derived classes to auxiliary information, in determining whether the best-fitted class-solution is empirically and theoretically meaningful^25^. Yet, while auxiliary variables (e.g., predictors or distal outcomes) can readily be incorporated in LVMM to validate the identified subgroups, evaluation and selection of the model is generally not determined by any external performance (e.g., prediction accuracy). Hence, subgroups identified through this approach may not provide useful prognostic information.

Both aforementioned concerns regarding the use of LVMM could potentially be combated within a machine learning framework. First, machine learning researchers have long acknowledged the susceptibility of highly flexible algorithms to overfitting the sample data; consequently, numerous approaches to both assessing and preventing overfitting (e.g., cross-validation) have been developed. Second, supervised learning methods incorporate an external criterion by which the classification model should be evaluated and optimized. In fact, LVMM has recently been formally integrated into a supervised learning framework with the main goal of evaluating candidate LVMM by linking them to clinically relevant auxiliary information^26^. In other words, rather than searching for the “true” model based on internal fit criteria, the primary purpose is to select a model based on its predictability. Indeed, accurately predicting or classifying individual-level outcomes is critical for enhancing the quality of personalized treatment and intervention. Thus, approaching subgrouping and pain classification from this prediction perspective may be preferable.

The main aim of the present study was to identify prognostically meaningful subgroups of chronic pain patients. Hence, the present study used a supervised machine learning framework (i.e., cross validation) for model selection and performance evaluation of LVMM. Specifically, with national real-world clinic data from chronic pain patients in Sweden, we identified models that capture individual heterogeneity in phenotypic characteristics using LVMM. Patients were grouped based on scores on several measures of pain characteristics, psychological aspects, and social functioning and general health status. For both model selection and performance evaluation, we used the self-reported pain interference one-year following the interdisciplinary treatment as the outcome; thus, by using this external criterion we could evaluate the empirically derived subgroups based on a direct measure of success (i.e., the predictability of the future outcome). To evaluate the expected accuracy of the selected model (i.e., class solution) on new data (i.e., unseen cases), we implemented a systematic nested cross-validation approach.

## Methods

### Participants and procedure

A sample of patients with complex chronic nonmalignant pain who were referred to specialist care centers from the Swedish Quality Registry for Pain Rehabilitation (SQRP) was used in this study. With Swedish national data, the SQRP provides important information from patients whose chronic pain is not caused by any other condition in need of urgent treatment^15^. The treatment received varies across clinical and patients in terms of both length and content: however, the treatment is typically delivered in a group format by an interdisciplinary team over several weeks to a few months, with a focus on pain education, supervised physical training and psychological interventions based on principles from cognitive behavioral therapy. Detailed description of the registry and collected data are presented elsewhere^15^.

During the data cleaning stage, we kept the cases with valid answers on follow-up pain intensity and interference, as well as removed 349 cases with duplicated identification numbers (potentially due to repeated registrations), resulting in a final subsample with 11,995 patients (*M*_age_ = 43.9 years; 77.6% women; 46% full-time employed; 82% Swedish born). The patients in this sample undertook their initial visits to a specialist unit for their pain issues from 2008 to 2016.

The baseline data were collected at the specialist unit on the patients’ initial visits. The follow-up data were subsequently accessed one year after the rehabilitation. All subjects and/or their legal guardian(s) gave their written and informed consent during their first visit. This study was approved by the Swedish Ethical Review Authority, 2018-036, EPM dnr: 2019-02167, EPM 2020-02038. It was performed in accordance with the Declaration of Helsinki.

### Measures

We selected our indicator variables mainly based on mainstream recommendations for pain treatments^2,27,28^ as well as findings from previous studies using SQRP for classification and prediction. The selected indicator variables comprise three domains: (1) pain characteristics, (2) psychological aspects, and (3) social functioning and general health status. The survey was undertaken in Swedish.

#### Indicator variables

##### Pain characteristics

Two widely used pain aspects from the Multidimensional Pain Inventory^29^ were used: *pain intensity* (2-item) refers to the magnitude of experienced pain, *pain interference* (11-item) reflects interference of pain in one’s work and social functioning. This inventory has shown good psychometric properties in previous Swedish studies^30^. The average score of each subscale represents the construct. With a 7-point scale (from *never* [0] to *very *often [6]), higher scores reflect stronger pain intensity, interference, and more life control, respectively. In addition, we also used the single-item *pain location number* (i.e., the number of anatomical regions with pain) as it appears to be a key correlate of pain aspects and indicates the pain spreading extent^31^. To quantify pain location numbers, a list of 36 predefined anatomical areas were presented to participants who then indicated their painful areas^31^. The number of pain locations ranged from 0 to 36, larger numbers indicate greater pain extent.

##### Psychological aspects

*Anxiety* and *depression* were included as previous studies suggest that these two variables are related to pain conditions as well as analgesic treatment outcomes^2^. We assessed these two constructs using the 14-item Hospital Anxiety and Depression Scale^32^, with a 4-point scale (e.g., from *not at all* [0] to *very often* [3]). The Swedish version^33^ has been found as a reliable instrument. Higher scores suggest stronger anxiety and depressive symptoms. The *affective distress* (3-item) from the Multidimensional Pain Inventory^29^ was also included as an indicator variable as it captures more general distress symptoms. Informed by the fear-avoidance model^34^, we further included *fear of movement*, which is considered as an anticipatory emotional response to a future bodily movement that could lead to painful feeling. Fear of movement was measured using the 17-item Tampa Scale for Kinesiophobia^35^, where each item is rated on a 4-point scale (from *strongly disagree* [1] to *strongly agree* [4]). A previous study^36^ showed the good reliability of this instrument among Swedish pain patients. Item scores were added up to form the total construct score; higher scores reflect more perceived fear of physical movements. In addition, *mental health* (5-item), a subscale from the Short Form Health Survey (SF-36)^37,38^ was included to reflect the overall mental health status.

##### Social functioning and general health status

Health-related quality of life has been suggested as another core criterion to assess in pain treatment^28^. Based on the Short Form Health Survey (SF-36)^37,38^, we specifically chose the overall *physical health* (21-item), which includes general health, physical functioning, role limitations due to physical problems, and body pain. The subscale *vitality* (4-item) was also included. While mental wellbeing has been suggested as a key assessment measure^28^, vitality has not been widely recommended as a key factor for analgetic outcome until recently^2^. Vitality plays an important role among pain patients as physical and mental energy is required to manage pain^39^. Items of each subscale were transformed and summed up as per the scale instructions^40^. Higher scores reflect more perceived vitality and physically healthier status, respectively. In addition, *life control* (4-item), which indicates how much control one generally has over their life, was selected from the Multidimensional Pain Inventory^29^.

#### Outcome variable

Two variables assessed at the one-year follow-up stage were used as distal outcome variables: (1) *pain interference (11-item)*, a subscale from the Multidimensional Pain Inventory^29^ and (2) *health status*, a single-item measure from the EQ-5D^41^. The pain interference scale properties are described above. For the health status measure, participants were asked to rate their general health status on a vertical analogue scale from 0 to 100, with 100 indicating the best possible health level. Pain interference was used in the cross-validation process because pain interference reflects overall impact of pain and serves as a primary concern of patients and represents a core outcome domain ^42,43^.

#### Background variables

To further understand the characteristics of each phenotype, we included sex, age, highest education level, employment status (full-time/part-time), visits to a physician about pain in the past year (0-1/2-3/over 4 times), pain duration (i.e., years of pain experience), and social support. Demographic and patient disposition factors were included as per previous recommendations^27,28^. As social support has been found as a key variable for pain recovery^18,20^, the social support subscale (2-item) from the Multidimensional Pain Inventory^29^ was used.

### Statistical analysis

#### Finite mixture model

The analytical model that was evaluated was a Gaussian finite mixture model. The model uses the information from the observed indicator variables to form subgroups where the grouping variable is formalized as a latent categorical variable with latent classes from ${c}_{1}$ to ${c}_{k}$. A mixture distribution is a weighted sum of $K$ component distributions as follows,

$$f\left({y}_{i}\right)={\sum }_{k=1}^{K}{\uppi }_{k}{f}_{k}({y}_{i}, {\theta }_{\text{k}})$$

where ${y}_{i}$ is a vector of observed indicator variables for individual $i$, ${\uppi }_{k}$ is a weight that quantifies the ${k}^{th}$ component’s relative size, and ${\theta }_{k}$ is a vector of model parameters for the ${k}^{th}$ component^44^. The component distributions, ${f}_{k}$, are the multivariate normal distributions, where the parameter vector ${\theta }_{k}$ contains the parameters with the class-specific means, ${\mu }_{k}$, and covariance matrices, ${\Sigma }_{k}$. The off-diagonal elements of ${\Sigma }_{k}$ matrices were fixed to zero (i.e., local independence assumption), and diagonal elements were constrained to be equal across classes. One can think of ${f}_{k}$ as the conditional probability of obtaining the observed sample data/indicator values for an individual from class $k$. Then, $f\left({y}_{i}\right)$ gives the unconditional probability for ${y}_{i}.$ We fitted a successively increasing number of classes, $k=\left\{2, 3, 4, 5, 6\right\}$. Models were evaluated with Mplus using maximum likelihood estimation and the EM-algorithm^45^. To avoid potential convergence at local maxima, we used random starting values (250 for the initial and 100 for the final stage optimization in Mplus). Posterior probabilities for each individual and class were computed based on model parameters $({\mu }_{k}, {\Sigma }_{k})$ and the observed individual score of each indicator variable [see equations 32 and 33^46^]. These computed probabilities were then used as predictors $(k-1)$ of the pain interference outcome at one-year follow-up, based on a linear regression model in the nested cross-validation procedure.

Although there are no formal tests for determining the assumption of normally distributed variables within each latent class in Gaussian finite mixture models, we visually examined the data distributions of each indicator for the whole sample and each class in the final class solution, finding that normality can be generally assumed across most distributions, although pain intensity, pain interference, and vitality seemed to have larger skewness.

#### Combined model for outcome prediction

Posterior class probabilities were computed for each individual in test sets of our cross validation and used for outcome prediction based on a linear regression model. The parameters for this regression model are obtained in the respective training set. This final prediction step constitutes the supervised part of our machine learning algorithm and hence enables constructing latent classes with the perspective of predicting a distal outcome.

#### Cross-validation for parameter optimization and performance assessment

**Figure 1:**
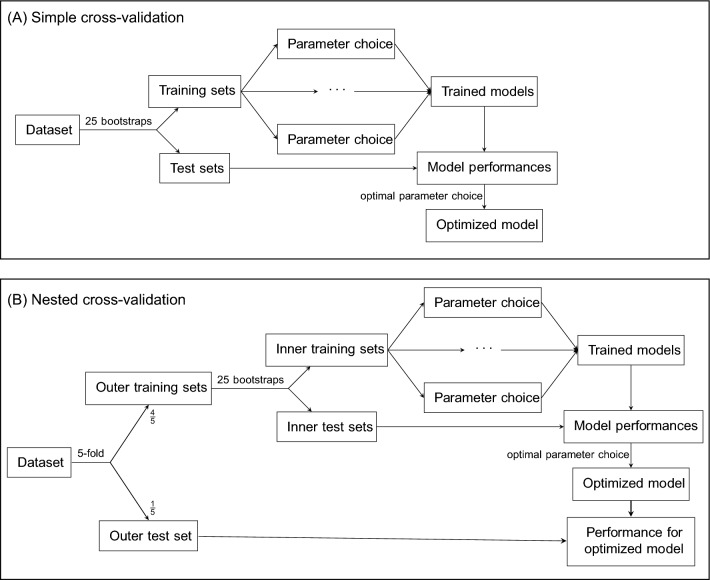
Workflow for two cross-validation schemes.

A cross-validation is performed whenever a model is fitted, to determine a suitable value for the parameters of the model, in our case the number $$k$$ of subgroups (or the number of subgroups, $$k)$$, based on prediction accuracy^47–49^. As detailed in Fig. 1A, a bootstrap approach is employed to repeatedly resample a training set from the data (predictors and outcome). Data not chosen during the bootstrap form the test set. Different parameter settings are applied to train a model for each round of the cross-validation, and each time the trained model’s performance is assessed using the test data. Model performance on these test set data was quantified as root mean squared error (RMSE). Finally, from average model performances for different parameter choices, the optimal parameter choice can be concluded. A final model is then fit to the entire dataset using this optimal parameter choice (i.e., yielding the optimized model).

Using a simple cross-validation, as described here above, a prediction model can be optimized, but its performance cannot be assessed at the same time. Therefore, we employed a nested cross-validation^50–52^, as shown in Fig. 1B: The available data are split into 5 parts, and 5 models are trained and optimized, each one leaving out one fifth of the data for measuring the performance. The other four fifths of the data (the outer training set) are split up further in the inner cross-validation (as in Fig. 1A). The performance of each optimized model can then be assessed using the share of the data that was left out in the training of the model, such that no sample is ever used to learn and evaluate the same model. Model performance on these outer test set data was again quantified as RMSE. Importantly, as it is not possible to fit any model to all available data, and, at the same time, estimate its performance on future new data, the average performance assessed in the five outer test-set predictions of our combined LVMM-linear models served as our best estimate for the future performance of such a “final” model. To be able to compare the results of this selection process to a conventional procedure for model selection we provide commonly used fit statistics used in mixture modeling in supplementary materials (Table S3).

Specifically, we applied a five-fold outer cross-validation in combination with a 25-fold bootstrap for the inner cross-validation. The R-package “MplusAutomation” (version 1.1.0)^53^ was used as an interface from R to Mplus (version 8.5)^54^. Our approach was then implemented as a custom model using the R-package “caret” (version 6.0.91)^55^, which provides the functionality for the inner cross-validation.

#### Subgroup characteristics and associations with auxiliary information

Based on the performance of models, the optimal latent class solution was then estimated using the whole dataset. The consequent subgroup profiling and comparisons were based on the optimal model (i.e., optimal $k$) selected from the cross-validation (as described above). Estimated means harvested from the whole sample-based model were plotted to profile the characteristics of each subgroup. To investigate how subgroup memberships related to both background variables and distal outcome (one-year follow-up pain interference), the three-step approaches were used in order not to allow the auxiliary information to influence the class-solution at this descriptive stage^56^. Effect sizes (Cohen’s d) for outcomes were computed when applicable.

Following the assumption of missing at random, the full information maximum likelihood approach was adopted for most analyses; three cases were removed since they had missing values on all indicators. Listwise deletion was used when examining the relationships between background covariates and class membership, with 9173 cases (76.5%).

## Results

### Cross-validation for parameter optimization and performance assessment

#### Model selection

**Figure 2:**
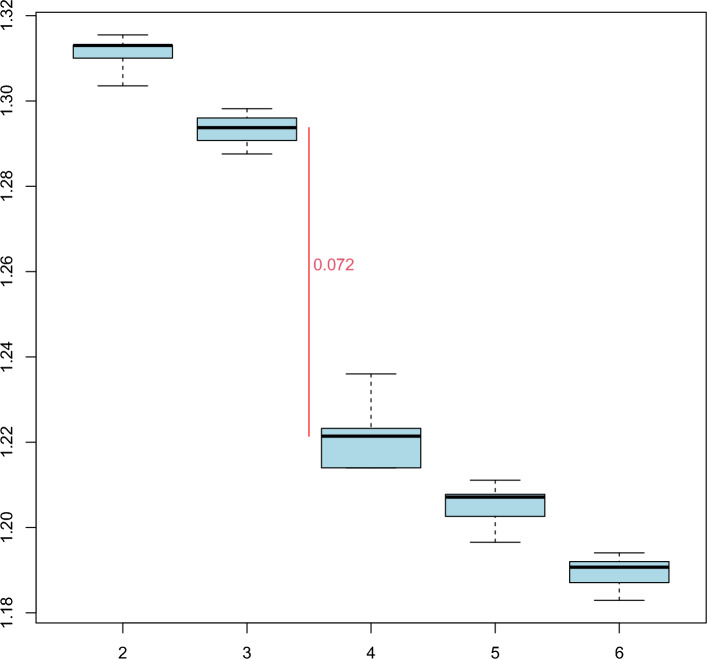
The Root-Mean-Square deviation (RMSE) deflection with four-class solution. *Note* The boxplots of the RMSE values (y-axis) for the five cross validations, for different subgroup numbers (x-axis). For each number of subgroups, the boxplots show the minimum, 1st, 2nd (median), 3rd quartiles, and the maximum of the RMSE values of the outer cross validations and their medians as horizontal black lines. The red vertical line denotes the maximum drop of the RMSE with reflected median difference between subgroup number 3 and 4.

Following the five-fold nested cross-validation, the optimal number of latent classes was chosen based on two- to six-class models in the inner cross-validation and their performances measured on the outer test sets. The resulting RMSE values of each model with respect to simplicity (of the model) and prediction of the outcome suggested the four-class model as the optimal (Fig. 2). Notably, there is one large relevant drop in RMSE values, from the three-class to the four-class model (∆_RMSE_ = 0.072). Similar trends were observed in other performance indices including R^2^ and mean square error; for brevity, these values are tabulated in supplementary materials (Table S1).

#### Model performance

The performance of the selected model $\left(k=4\right)$ was evaluated in five outer cross-validation rounds as five-fold cross-validation, such that each sample belonged to one of the five outer test sets and was predicted once by a model which was trained on 80% of the total data. As assessed with a linear regression, the predicted pain interference outcome value by the latent classes (i.e., posterior probabilities) captured about 26% of the variation in the true pain interference values of the dataset. Of note, performance of the combined LVMM-linear model was evaluated by (outer) cross-validation, namely, using the fitted model to predict the outcome for unseen data (i.e., the five outer test sets comprising 20% of all data each which have not been used for model selection purpose).

### Optimized final mixture model fitted to all data

#### Profile of subgroups

**Figure 3:**
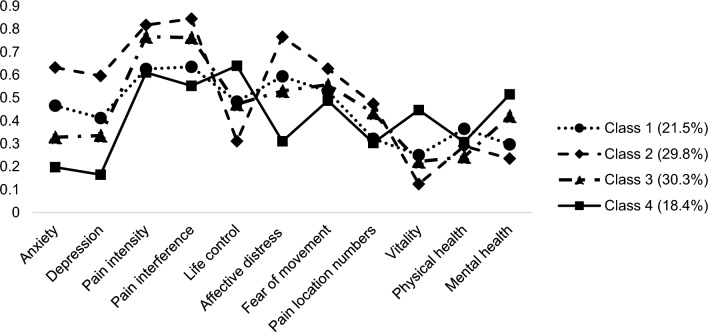
Estimated means of the four-class model generated from latent variable mixture modeling. *Note* To visually compare between indicators with various ranges, estimated means ($\overline{x }$) of each indicator have been rescaled based on the theoretical maximum value (${max}_{x}$) of each scale (i.e., $\frac{\overline{x}}{{max }_{x}}$). A larger rescaled score reflects a higher rank of an estimated mean.

Informed by the previous steps, an LVMM with $k=4$ was performed in the whole sample, resulting in four qualitatively distinct subgroups. Figure 3 visually illustrates the characteristics of subgroups (for tabulated information, see Table S2 in supplementary materials).

Class 1 comprised 2573 patients (21.5%) who reported relatively low pain intensity, interference, and pain location numbers. Although Class 1 members showed the best level of general physical health, their negative emotions such as anxiety and depression were notably higher.

With 3575 patients (29.8%), Class 2 demonstrated the worst levels on most indicators. Specifically, members of Class 2 reported severest pain intensity, interference, as well as pain extent. Their anxiety, depression, and general affective distress were also highest among the subgroups. Moreover, these patients had markedly lower mental health and vitality scores.

Class 3 was the largest subgroup (30.3%), with 3638 patients. Patients in Class 3 had the lowest level of physical health, although their negative emotions were relatively moderate compared with other subgroups. Even though Class 3 patients showed a similarly high level in pain intensity, interference, and pain extent as in Class 2, they had a relatively more life control than Class 2 members.

Class 4 (*n* = 2206; 18.4%) showed the best scores on most indicators. Their pain presentation and negative emotions were the lowest, with a better health condition especially mental health and vitality. Based on these subgroup features, Class 4 was used as the reference group in the subsequent analyses as it is regarded as the heathiest subgroup.

#### Subgroup associations with follow-up outcomes

**Figure 4:**
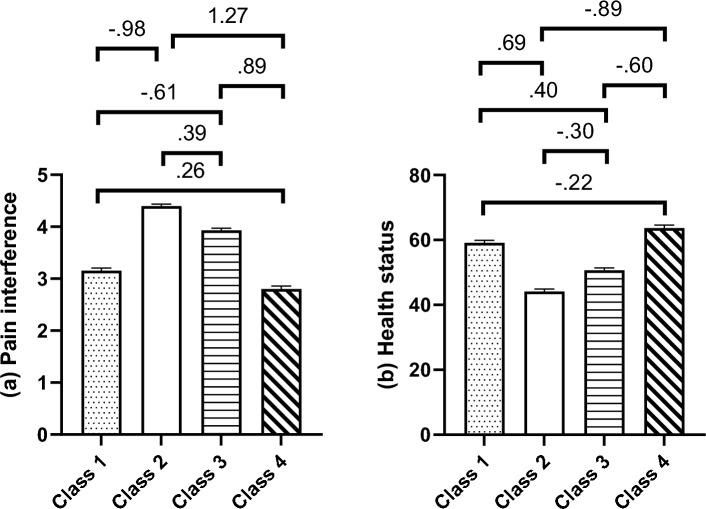
Mean scores of distal outcome variables assessed at one-year follow-up. *Note* Mean score with 95% confidence interval is presented. Only the above part of error bar is displayed to avoid visual clutters. Numbers over the zig–zag lines indicate the effect size (Cohen’s *d*) of pairwise comparisons.

With the three-step approach which included distal outcomes, the between-subgroup difference in the follow-up pain interference (χ^2^_(3)_ = 3545.30, *p* < 0.001) and health status (χ^2^_(3)_ = 1172.73, *p* < 0.001) both showed an overall statistical significance. Figure 4 shows the means for each subgroup and the standardized mean differences (Cohen’s d) for all group comparisons.

For pain interference, all between-subgroup differences were statistically significant (*p*s < 0.001) except for the comparison between Classes 1 and 4 (*p* = 0.130). Large effect sizes (*d* ≥ 0.80) were found in Class 2 vs. Class 4 (*d* = 1.27), Class 1 vs. Class 2 (*d* =  − 0.98), and Class 3 vs. Class 4 (*d* = 0.89).

For health status, all between-subgroup differences showed a statistical significance (*p*s < 0.001; Class 1 vs. Class 4, *p* = 0.001). Large effect size (*d* ≥ 0.80) was found in Class 2 vs. Class 4 (*d* =  − 0.89).

#### Subgroup associations with background characteristics

Table 1 descriptively reports the background variables of the four subgroups based on the most likely class membership of each subject (i.e., largest posterior probabilities). When background variables were included as auxiliary predictors, all variables showed associations with the class membership (Table 2). Compared with the healthiest subgroup (Class 4), other classes were younger. Overall, all subgroups had more women than men (i.e., > 70% women); between-subgroup differences were found in sex distributions. Patients in Classes 2 and 3 had more women and were less educated than Class 4, whereas those in Class 1 were more educated than Class 4. Furthermore, as opposed to Class 4, members in Classes 2 and 3 were less full-time employed, had more visits to a pain clinic in the past year, as well as had longer pain durations. While patients in Class 1 showed less social support than Class 4, Class 3 members had more social support than Class 4 members. In addition, Class 3 members perceived more social support than Class 4 members, whereas Class 1 members perceived less.

**Table 1:** Descriptive statistics of background variables by classes.

Variable-frequency	Class 1	Class 2	Class 3	Class 4
Women–%	75.8%	79.6%	81.2%	73.8%
Age–*M* (years)	42.9	43.1	44.2	45.8
Highest education level^a^				
Primary	250 (10.7%)	534 (16.0%)	466 (13.7%)	224 (11.0%)
Secondary	1248 (53.6%)	1988 (59.4%)	1982 (58.4%)	1140 (55.9%)
Tertiary	829 (35.6%)	824 (24.6%)	945 (27.9%)	675 (33.1%)
Full-time employment–%	56.4%	39.3%	45.4%	58.8%
Visits to a pain clinic in the past year^a^				
0–1 times	310 (13.2%)	152 (4.5%)	214 (6.3%)	292 (14.0%)
2–3 times	682 (29.0%)	568 (16.7%)	767 (22.4%)	643 (30.8%)
≥ 4 times	1361 (57.8%)	2688 (78.9%)	2443 (71.3%)	1156 (55.3%)
Pain duration (years)–*M* (*SD*)	8.1 (9.1)	8.5 (9.2)	8.6 (9.0)	8.6 (9.9)
Social support–*M* (*SD*)^b^	3.74 (1.38)	4.18 (1.42)	4.40 (1.23)	4.14 (1.28)

^a^Percentages show the proportions in each Class (i.e., column percentages).

^b^The theoretical range of social support is between 0 and 6.

**Table 2:** The logit coefficients of predictors from the three-step approach.

Variable	Class 1	Class 2	Class 3
Sex	− 0.05	0.53***	0.67***
Age	− 0.03***	− 0.02***	− 0.01***
Higher education level	0.15*	− 0.27***	− 0.18**
Full-time employment	− 0.11	− 0.79***	− 0.60***
Visits to a pain clinic in the past year	0.10	0.93***	0.60***
Pain duration	0.00	0.01**	0.01**
Social support	− 0.27***	0.00	0.20***

Class 4 is the reference group. With listwise deletion, 9173 cases (76.5%) with complete data were used for the above regression estimation. Sex: 1 = man, 2 = woman. Educational level: 1 = primary education, 2 = secondary education, 3 = tertiary education. Full-time employment: 1 = yes, 0 = no. Visits to a pain clinic in the past year: 0 = 0-1 time, 1 =2–3 times, 2 = over 4 times. Pain duration: years of pain experience. Social support: a subscale from the Multidimensional Pain Inventory [^29^, with higher scores meaning more perceived social support for their pain problems.  *p < .05. **p < .01. ***p < .001.

## Discussion

Our study aimed to identify prognostically meaningful subgroups of chronic pain patients using data from a naturalistic and population level setting. In contrast to previous work that used unsupervised clustering approaches, a supervised machine learning framework was used for model selection and performance evaluation. Based on the optimal solution, four qualitatively distinct subgroups of chronic pain patients were discerned. The four-class solution showed a robust explanatory ability (R^2^ = 26%) of the long-term follow-up on unseen data in the outer cross-validation, providing support for the predictive validity of derived subgroups using a rigorous approach for performance evaluation. This means that a significant part of the observed variation in pain outcome can be explained by a simple linear model of class-membership posterior probabilities estimated for each subject of the test-sets in our cross-validation. This was corroborated by effect size differences of non-trivial magnitude on pain interference and general health status at one-year follow-up between specific subgroups when the final model was fitted to the whole sample. The subgroups were also distinguishable based on examined background characteristics. Thus, as a novel method to combine an advanced unsupervised person-centered analytical method (i.e., LVMM) with a supervised learning approach to classification, our initial findings are encouraging, especially given that previous studies have generally failed to identify reliable predictors of long-term outcomes following interdisciplinary treatment using simpler regression- and variable-centered analytical approaches^11^.

In agreement with both mainstream recommendations and growing body of evidence^27,28^, our results show that psychological and pain characteristics (e.g., pain intensity) are both important phenotypic characteristics that interact at the level of the individual. The four subgroups identified in our study have their distinct characteristics. Class 4 (18.4%), with relatively low scores on pain characteristics, negative emotions, and better health status, appeared to be the healthiest subgroup. In contrast, with elevated levels of pain characteristics and negative emotions as well as poorer health status, Class 2 (29.8%) was an ill and sizable subgroup. Classes 1 (21.5%) and 3 (30.3%) showed a medium level on all indicators, although nuanced differences are observed between these two subgroups: Class 1 had higher anxiety, depression, and affective distress than Class 3, although members of this Class had less pain symptoms and better health status than Class 3.

These subgroups identified in our study have certain similarities with previous typologies of pain patients discovered in studies using various clustering methods. In a seminal study which also included both pain characteristics and psychological variables^20^, three subgroups among chronic pain patients were named as “dysfunctional”, “interpersonally distressed”, and “adaptive copers”. This three-subgroup taxonomy is comparable with Class 2, Class 1/3, and Class 4 in our study. Our findings are also in broad agreement with recent studies that have aimed to identify subgroups in patients receiving interdisciplinary treatment. Most relevant, a study using LVMM^16^ also identified four similar subgroups of patients in the degree of pain severity, physical health, and emotional distress. While this study^16^ relied on a conventional, exploratory approach to determining the number of subgroups, findings, taken together, support the four latent classes among chronic pain patients across samples.

A noteworthy finding, with potential clinical implications, is that two of the classes (Classes 1 and 2) had patients with marked emotional distress (anxiety and depression). As noted^7^ after reviewing pain patient phenotype studies, research tends to discern a subgroup with greater pain intensity and negative emotions, highlighting the central phenotypic role of mental health status. Consistent with a substantial body of evidence supporting bi-directional links between general negative affect and severity of pain^2^, as well as with findings from previous studies on patient types^6,57^, increased negative emotions and pain presentations appear to cooccur. Specifically, the increased anxiety and depression observed in Class 2 (with the worse conditions across most indicators) highlight the key role of negative emotions among those individuals with worse clinical presentation at intake as well as increased risk of unfavorable long-term outcomes. In this context, the interrelationships between fear of movement, anxiety, and depression are also worth noting. A previous clustering study^6^ found two clusters with similar levels of fear of movement, but one of these patient clusters showed distressed mood, as well as higher pain intensity.

The additive effect of emotional distress on both pain and fear may necessitate targeted interventions. Indeed, more recently, the importance of interventions targeting emotional processing and regulation have been highlighted for specific subgroups of chronic pain patients with high emotional distress^58,59^—treatment approaches that may be particularly valuable for Classes 1 and 2. In comparison to Class 1, Class 2 members showed elevated symptoms on all indicators, suggesting the need for a more comprehensive interdisciplinary treatment in targeted interventions. As previously recommended^6^, Class 2 members may also benefit specifically from exposure-based treatment given their high fear of movement. While specific treatment recommendations are in need of confirmatory tests, our findings call into question the one-size-fits-all approach to pain treatment and highlight the importance of identifying subgroups for improved treatment matching, which has long been recognized in the field^5^.

The associations between covariates and subgroup membership provide further information about the latent classes. A previous study^16^ has identified a subgroup, which had lower scores on pain characteristics, but slightly more negative emotions; this subgroup members were remarkably older than others (10–15 years). Similar to this subgroup, Class 4 in our study, with less pain and negative emotions, was also older than other subgroups. It is important to note that Class 4 members also received more education and had less pain duration than those in Classes 2 and 3. An interesting contrast among groups was observed in social support. Compared to Class 4, the healthiest subgroup, Class 1 patients perceived less social support whereas Class 3 members perceived more. In previous subgroup studies, lack of social support has been found to be associated with being in subgroups which showed longer pain duration^17^ or severer pain burden^16^. It may partially explain why Class 3 members in our study showed relatively lower levels of negative emotions, even though their pain intensity was similar to Class 2. In contrast, with lower social support, Class 1 members had more emotional distress than Classes 3 and 4, although its pain intensity was similar to Class 4. Our findings indicate that it is useful to consider the relationship between pain and emotional distress with the consideration of social support.

By combining the strengths of unsupervised and supervised learning approaches, our study also offers a novel methodological perspective on subgrouping in pain research. The framework expands on a recent methodological approach in which the selection of mixture model is governed by its prediction accuracy^26^. Using a nested cross-validation procedure, we were able to use an analgesic outcome in the selection of model as well as independently estimating the effects of the selected model for unseen cases. Replicability of clusters has always been a key focus because it indicates how reliable the cluster profiling is across samples^20,60–62^. However, failure to replicate different class solutions has been a source of concern, as has the ability to use subgroups for prognostic and treatment matching purposes. To date, simpler cross-validation procedures in which the sample data is split in to an exploratory and confirmatory part have been utilized to combat overfitting and replication issues in mixture modeling^23^. By combining a highly flexible unsupervised method with nested cross-validation and an optimization based on a supervised prediction of a one-year follow-up outcome, our study has continued this rigorous lineage.

While our statistical learning approach is promising for identifying prognostically meaningful subgroups, there are multiple ways to modify the analytical framework and widen its application. First, both the selection of indicators and the weights of chosen indicators could also have been optimized for outcome prediction using an appropriate supervised method prior to formation of latent classes in mixture modeling. Second, a single outcome variable was used as the target and a simple linear regression model was used to predict outcome from posterior class-membership probabilities; by using several outcomes and examining non-linear relations with outcomes could provide a more comprehensive evaluation of the models. Third, there are ways in which the finite mixture models could have been optimized further, including, for example, exploring additional individual heterogeneities within each class by combining mixture modeling with factor analysis (factor mixture model)—a method that has shown promise in identifying subgroups in specific pain conditions^24^. Finally, Gaussian mixture modeling assumes normally distributed variables within each latent class and when this assumption is violated can create spurious class solutions. However, recent methodological developments have extended mixture model to handle non-normal data reducing the risk of extracting classes that are merely due to non-normality^63^. Nonetheless, a key strength of LVMM is that the framework enables empirical comparisons of a diverse set of models (e.g., growth mixture models, factor mixture models)–all done within one coherent analytical framework. Future research may build on this combined framework of unsupervised and supervised learning to incorporate information from additional sources (e.g., variables collected during treatment), examine individual change trajectories, and treatment response patterns in randomized experiments to identify clinically meaningful subgroups. Regardless of mixture model and applied context, the framework enables the identification of prognostically meaningful subgroups based on their ability to predict outcomes.

Our study has several limitations. The rehabilitation treatment varied across clinics and patients were not randomized to different treatments; therefore, the causes for outcome variations at follow-up remain unknown. Only patients with follow-up pain outcomes were included in this study; the reasons for non-adherence are not known, potentially biasing estimates due to non-ignorable causes of missing data. While guidelines about phenotyping pain patients are available, researchers have employed various strategies to select indicators. In our study, we focused on a restricted number of indicators and biological information such as genetic data and specific diagnosis were not included in the phenotypic characteristics. Moreover, social domains in our study are not fully represented. A considerable number of previous studies investigate subgroups among a specific type of pain patients (e.g., fibromyalgia, low back pain). Our study assumed that psychological aspects, self-perceived pain, and health status were independent of physical pathology across various types of pain conditions^18^. However, the assumption was not tested and nor did our study measure patients’ beliefs about etiology, which have been related to recovery process^64^.

Notwithstanding, our study utilized a large, national sample follow-up assessments among chronic pain patients, mitigating limitations identified in previous pain patient classification studies. Using a rigors approach for identifying and evaluating our analytical model, our findings add to growing body of studies aiming to identify clinical meaningful subgroups across diverse chronic pain patients. From an applied methods standpoint, our study provides a novel person-centered supervised machine learning analytical framework that can be further expanded upon to ultimately provide an empirically derived taxonomy for treatment matching and prediction.

### Supplementary Information

Supplementary Information.

## Data Availability

The datasets generated and/or analyzed during the current study are not publicly available due to privacy or ethical restrictions but are available from the corresponding author on reasonable request.
