# Supplementary material for: Prognostic subgroups of chronic pain patients using latent variable mixture modeling within a supervised machine learning framework
Source: Sci Rep. 2024 May 31;14:12543. doi: 10.1038/s41598-024-62542-w (PMC11143186; doi:10.1038/s41598-024-62542-w)
Supplement: Supplementary file 1 — Supplementary Information. [file 41598_2024_62542_MOESM1_ESM.docx]

**Supplementary materials**

Table S1

*Original Root-Mean-Square Deviation in All Five Rounds*

| Solution | Round 1 | Round 2 | Round 3 | Round 4 | Round 5 |
| --- | --- | --- | --- | --- | --- |
| 2-class | 1.313005 | 1.313054 | 1.303532 | 1.315484 | 1.310023 |
| 3-class | 1.29373 | 1.29602 | 1.287578 | 1.298167 | 1.290727 |
| 4-class | 1.213966 | 1.23601 | 1.214006 | 1.221422 | 1.223264 |
| 5-class | 1.2026 | 1.211098 | 1.196559 | 1.207808 | 1.207126 |
| 6-class | 1.187088 | 1.190717 | 1.182945 | 1.194058 | 1.192014 |

Table S2

*Four Subgroups with Estimated Mean and Standard Error of Each Indicator*

| Indicator | Class 1 | | Class 2 | | Class 3 | | Class 4 | |
| --- | --- | --- | --- | --- | --- | --- | --- | --- |
|  | M | SE | M | SE | M | SE | M | SE |
| Anxiety | 9.79 | 0.14 | 13.28 | 0.13 | 6.90 | 0.33 | 4.16 | 0.09 |
| Depression | 8.66 | 0.17 | 12.53 | 0.12 | 7.05 | 0.28 | 3.47 | 0.08 |
| Pain intensity | 3.76 | 0.14 | 4.91 | 0.03 | 4.60 | 0.03 | 3.67 | 0.06 |
| Pain interference | 3.81 | 0.17 | 5.07 | 0.03 | 4.58 | 0.03 | 3.32 | 0.07 |
| Life control | 2.90 | 0.04 | 1.87 | 0.04 | 2.84 | 0.04 | 3.84 | 0.04 |
| Affective distress | 3.56 | 0.05 | 4.60 | 0.03 | 3.18 | 0.09 | 1.86 | 0.04 |
| Fear of Movement | 35.96 | 0.64 | 42.67 | 0.30 | 37.91 | 0.27 | 33.18 | 0.31 |
| Pain Location Numbers | 11.60 | 0.47 | 17.07 | 0.24 | 15.64 | 0.27 | 10.94 | 0.29 |
| Vitality | 25.08 | 0.86 | 12.42 | 0.39 | 22.24 | 0.53 | 44.72 | 1.02 |
| Physical health | 36.53 | 1.19 | 28.98 | 0.29 | 24.31 | 0.72 | 30.71 | 0.63 |
| Mental health | 29.73 | 0.52 | 23.53 | 0.25 | 42.12 | 1.33 | 51.53 | 0.30 |

Note. M = Estimated mean. SE = Standard error.

Table S3

*Fit Indices for Models from Two (C2) - to Six-Class (C6)*

|  | AIC | BIC | SABIC | Entropy | Adj. LMR-LRT | Smallest subgroup size (%) |
| --- | --- | --- | --- | --- | --- | --- |
| C2 | 638357.69 | 638609.01 | 638500.97 | 0.83 | 26582.25 (*p* < .001) | 46.7% |
| C3 | 629688.78 | 630028.82 | 629882.63 | 0.82 | 8616.45 (*p* < .001) | 22.9% |
| C4 | 625106.11 | 625534.85 | 625350.53 | 0.79 | 4566.16 (*p* = .008) | 18.4% |
| C5 | 621527.91 | 622045.34 | 621822.89 | 0.81 | 3570.52 (*p* < .001) | 9.9% |
| C6 | 619389.08 | 619995.22 | 619734.63 | 0.81 | 2143.81 (*p* = .161) | 2.7% |

Note. AIC = Akaike information criterion. BIC = Bayesian information criteria. SSABIC = Sample size adjusted BIC. Adj. LMR-LRT = Adjusted Lo-Mendell-Rubin likelihood ratio test.
